# ADAMTS-13 activity in stroke of known and unknown cause: Relation to vascular risk factor burden

**DOI:** 10.3389/fneur.2022.1045478

**Published:** 2023-01-10

**Authors:** Gerrit M. Grosse, Andrei Leotescu, Jan-Thorben Sieweke, Sonja Schneppenheim, Ulrich Budde, Nora L. Ziegler, Saskia Biber, Maria M. Gabriel, Johanna Ernst, Ramona Schuppner, Ralf Lichtinghagen, Udo Bavendiek, Julian Widder, Karin Weissenborn

**Affiliations:** ^1^Department of Neurology, Hannover Medical School, Hannover, Germany; ^2^Department of Cardiology, Hannover Medical School, Hannover, Germany; ^3^Medilys Laboratory, Asklepios Klinik Altona, Hamburg, Germany; ^4^Institute of Clinical Chemistry, Hannover Medical School, Hannover, Germany; ^5^Medizinische Klinik VI, Kardiologie, Angiologie und Internistische Intensivmedizin, Städtisches Klinikum Karlsruhe, Karlsruhe, Germany

**Keywords:** ADAMTS-13, biomarker, embolic stroke of undetermined source, patent foramen ovale, stroke etiology, thrombosis

## Abstract

**Background:**

The identification of the underlying mechanism in ischemic stroke has important implications for secondary prevention. A disintegrin and metalloprotease with a thrombospondin type 1 motif, member 13 (ADAMTS-13) has antithrombotic properties and was repeatedly implicated in the pathophysiology of stroke. In this study, we, therefore, aimed to investigate whether ADAMTS-13 is associated with stroke etiology and the burden of vascular risk factors.

**Methods:**

We determined ADAMTS-13 activity in two prospectively recruited stroke cohorts in the long-term course after the event. Cohort 1 (*n* = 88) consisted of patients who suffered a stroke due to embolic stroke of undetermined source (ESUS), cardioembolic stroke due to atrial fibrillation (AF), large-artery atherosclerosis, or small vessel disease. In cohort 2, patients with cryptogenic stroke and patent foramen ovale (PFO) scheduled for PFO closure (*n* = 38) were enrolled. As measures of vascular risk factor burden, the CHA_2_DS_2_VASC score, the Essen Stroke Risk Score (ESRS), and the Risk of Paradoxical Embolism (RoPE) score were calculated, as appropriate.

**Results:**

ADAMTS-13 activity was lower in patients with AF-related stroke compared to patients with ESUS (*p* = 0.0227), which was, however, due to confounding by vascular risk factors. ADAMTS-13 activity inversely correlated with the ESRS (*r* = −0.452, *p* < 0.001) and CHA_2_DS_2_VASC (*r* = −0.375, *p* < 0.001) in cohort 1. In accordance with these findings, we found a positive correlation between ADAMTS-13 activity and the RoPE score in cohort 2 (*r* = 0.413, *p* = 0.010).

**Conclusion:**

ADAMTS-13 activity is inversely correlated with the number of vascular risk factors across different stroke etiologies. Further study is warranted to establish ADAMTS-13 as a mediator of cerebrovascular risk.

## Introduction

Embolic stroke of undetermined source (ESUS) describes a cryptogenic stroke with embolic pattern on imaging and accounts for ~17% of all ischemic strokes (1). It is assumed that a relevant part of ESUS is due to an inapparent cardioembolic mechanism including atrial cardiopathy (2, 3) and atrial fibrillation (AF) (1). However, two randomized controlled trials did not show a superiority of oral anticoagulants over antiplatelet therapy in ESUS (4), implying that a proper work-up for identifying the most probable mechanism of stroke etiology is still warranted in patients who suffered a cryptogenic stroke. In addition to atrial cardiopathy, potential embolic sources in ESUS include non-stenotic carotid plaques (5, 6), aortic atherosclerosis (7, 8), cardiac valvulopathies, coagulopathies or paradoxical embolism *via* patent foramen ovale (PFO), and others (9). Interestingly, in a sub-analysis of the NAVIGATE-ESUS trial, Ntaios et al. showed that ~40% of patients with ESUS revealed multiple potential embolic sources (10). This finding was also confirmed in a large real-world sample of patients with ESUS (11). This evidence thus, furthermore, underscores the relevance to elucidate the most likely cause, especially since the underlying mechanism in ESUS also seems to have implications for functional stroke outcomes (12). However, there is still limited evidence on the individual risk of recurrence in patients with ESUS (13), whereas it has been shown that the CHA_2_DS_2_VASC score can provide a sound estimate of recurrence at the population level (14). Of note, most recurrent strokes in patients with ESUS again are embolic and cryptogenic (15).

Biomarkers might be useful to assist in the diagnosis of the underlying mechanism, to estimate individual vascular risk, and finally to guide the choice of antithrombotic treatment in patients who suffered ESUS (16). A disintegrin and metalloprotease with a thrombospondin type 1 motif, member 13 (ADAMTS-13) degrades ultra-large von Willebrand factor (VWF) multimers and therefore has antithrombotic properties. Severely reduced activity of ADAMTS-13 is the hallmark in the pathogenesis of thrombotic thrombocytopenic purpura (TTP) (17). Furthermore, ADAMTS-13 was repeatedly implicated in the pathophysiology of ischemic stroke (18) and was shown to be associated with response to reperfusion therapies of acute cerebral ischemia (19). Interestingly, low ADAMTS-13 activity was also identified as an independent risk factor for pediatric stroke (20). Thus, it is reasonable to hypothesize that ADAMTS-13 may have a role in the pathophysiology of cryptogenic stroke *via* its regulatory role in arterial thrombogenicity. Moreover, lower ADAMTS-13 activity was also implicated in the risk for deep vein thrombosis (DVT) (21), which may be of interest in the mechanisms of paradoxical embolism in stroke patients with PFO.

In this study, we, therefore, aimed to investigate whether ADAMTS-13 may be a circulating biomarker that supports the identification of the underlying mechanism of ischemic stroke. We enrolled two different stroke cohorts in which we determined ADAMTS-13 activity in peripheral venous blood in the long-term course after the event. Cohort 1 consisted of patients who suffered a stroke due to ESUS, AF, large-artery atherosclerosis (LAA), or small vessel disease (SVD). In cohort 2, patients with cryptogenic stroke and patent foramen ovale (PFO) scheduled for PFO closure were considered. We hypothesized that (I) ADAMTS-13 activity differs according to stroke etiology, (II) ADAMTS-13 activity is inversely associated with the number of vascular risk factors, and (III) lower ADAMTS-13 activity may reflect a higher probability for paradoxical embolism in stroke patients with PFO.

## Methods

We prospectively recruited two cohorts of patients with stroke who were treated in the Departments of Neurology and Cardiology at Hannover Medical School. Cohort 1 was initially recruited between August 2016 and April 2017 (22, 23) and underwent a subsequent long-term follow-up between August 2017 and April 2018 (24). As described previously (24), patients were assigned to four groups according to stroke etiology, that is, ESUS, cardioembolic stroke (CES) due to AF, LAA, and SVD. ESUS was defined according to the RESPECT-ESUS trial, as described previously (24). The exclusion criteria were rare stroke etiologies, current malignant diseases, and evidence of DVT in combination with PFO. Standardized stroke diagnostics were available for all patients, including cerebral and cranial imaging (MRI or CT, and MRI or CT angiography), Doppler and duplex ultrasound of the brain supplying arteries, and echocardiography. Prolonged cardiac monitoring scheduled for 72 h was realized for patients without known AF at baseline. Additional cardiac monitoring using Holter-ECG during follow-up was documented. A standardized case report form (CRF) was used to document clinical and demographical characteristics. Peripheral venous blood taken at follow-up was considered for this investigation. Serum samples were stored at −80°C until ADAMTS-13 measurements. To sum up vascular risk factors, the CHA_2_DS_2_VASC score and the Essen Stroke Risk Score (ESRS) were calculated. The ESRS was previously shown to accurately stratify stroke risk in patients without AF (25), while the CHA_2_DS_2_VASC is validated to provide information on stroke risk in AF (26).

Cohort 2 was prospectively recruited between July 2019 and July 2021. The inclusion criteria in cohort 2 were ischemic stroke and evidence of a PFO with an indication for PFO closure. The exclusion criteria were stroke without evidence in cerebral imaging, that is, magnetic resonance imaging (MRI) or computed tomography (CT), and stroke of other defined origin. Clinical and demographical data were obtained using a standardized CRF. Peripheral venous blood was drawn before PFO closure, and citrate plasma was stored at −80°C until ADAMTS-13 measurements. The Risk of Paradoxical Embolism (RoPE) score was calculated for all patients (27), as well as CHA_2_DS_2_VASC and ESRS. Cases in cohort 2 were assigned to a group with high (RoPE 6–10) or a group with lower PFO-attributable risk (RoPE 0–5). For all patients in both cohorts, the National Institutes of Health Stroke Scale (NIHSS) and the modified Rankin Scale (mRS) were obtained at the time of blood collection.

ADAMTS-13 activity was determined using the Technozym ADAMTS-13 ELISA (Technoclone, Wien, Austria).

The ethics committee at Hannover Medical School consented to the study (Ethics vote Nos. 3316-2016 and 8394_BO_S_2019). All patients provided written informed consent before being enrolled. The study was conducted in accordance with the Declaration of Helsinki.

Group differences of continuous data were analyzed using the two-sided Student's *t*-test for normally distributed data or the Mann–Whitney *U*-test for ordinal or non-normally distributed data. Categorical data were analyzed using the chi-square test or Fisher's exact test, as appropriate. Correlations between ADAMTS-13 activity and risk scores were calculated using Spearman's correlation. Binary logistic regression analysis was done to estimate the association between ADAMTS-13 activity and study groups in cohort 1 adjusting for the CHA_2_DS_2_VASC score. This was done based on the evidence that vascular risk factor burden is more emphasized in stroke patients with AF compared to patients with ESUS. Moreover, the CHA_2_DS_2_VASC was described as a risk factor for newly identified AF after stroke (28). The area under the receiver operating characteristic curve (AUROC) was calculated for evaluating the diagnostic accuracy in discriminating AF from ESUS. Linear regression analysis was performed to estimate the effect of distinct vascular risk factors on ADAMTS-13 activity in both cohorts. Boxplots depict boxes with Tukey's whiskers. Statistical analyses were done using IBM SPSS Statistics 26 (IBM SPSS, Armonk, NY, USA) and SAS Enterprise Guide 7.1 (SAS Institute Inc., Cary, NC, USA). Figures were created using GraphPad Prism 9 (GraphPad Inc., La Jolla, CA, USA).

## Results

Tables 1, 2 show the demographical and clinical characteristics of both cohorts. In cohort 1, in 18 patients, AF was newly diagnosed during the follow-up. Of note, age and the number of vascular risk factors significantly differed between stroke etiologies in cohort 1. In cohort 2, as expected, the total burden of vascular risk factors was lower than in cohort 1 and patients were younger, in accordance with the current recommendations regarding PFO closure after stroke. The median time from index event to blood sampling was 385 days in cohort 1 and 102 days in cohort 2.

**Table 1:** Clinical and demographical data of cohort 1.

	**ESUS (*n* = 42)**	**CES (*n* = 21)**	**LAA (*n* = 8)**	**SVD (*n* = 17)**	***P*** **-value**
Age (years)	70.50 (53.75–77.25)	80.00 (76.50–83.50)	66.50 (62.50–74.50)	67.00 (61.00–79.50)	0.001
Sex (female)	18 (43%)	7 (33%)	3 (38%)	6 (35%)	0.927
Arterial hypertension	27 (64%)	20 (95%)	8 (100%)	15 (88%)	0.007
Diabetes	10 (24%)	4 (19%)	1 (13%)	2 (12%)	0.756
Adiposity	9 (21%)	3 (14%)	2 (25%)	5 (29%)	0.716
Dyslipoproteinemia	24 (57%)	12 (57%)	5 (63%)	13 (76%)	0.580
Peripheral artery disease	4 (10%)	1 (5%)	2 (25%)	0 (0%)	0.163
Nicotine consumption	28 (67%)	11 (52%)	7 (88%)	11 (65%)	0.349
Alcohol consumption	7 (17%)	2 (10%)	4 (50%)	7 (41%)	0.022
Coronary artery disease	2 (5%)	4 (19%)	2 (25%)	1 (6%)	0.109
Myocardial infarction	2 (5%)	1 (5%)	1 (13%)	1 (6%)	0.817
Atrial fibrillation	0 (0%)	21 (100%)	0 (0%)	0 (0%)	< 0.001
Pos. Family history	16 (38%)	4 (19%)	1 (13%)	6 (25%)	0.322
NIHSS at enrollment	0 (0–1)	0 (0–1)	0 (0–1.5)	0 (0–2)	0.629
mRS at enrollment	1 (0–1.25)	2 (0–2.50)	1 (0–1.75)	1 (0–2.50)	0.812
BMI (kg/m^2^)	27 (23–29)	26 (24–30)	24 (23–32)	27 (25–32)	0.596
CHA_2_DS_2_VASC	4.50 (3.00–5.00)	5.00 (5.00–6.00)	4.50 (4.00–5.75)	4.00 (3.50–5.00)	0.005
ESRS	3.00 (2.00–4.00)	4.00 (4.00–6.00)	3.50 (2.25–4.75)	3.00 (2.50–4.00)	0.012
Time from index event to follow-up (days)	392 (373–454)	386 (377–417)	375 (371–444)	378 (367–424)	0.444

Continuous data are presented as median (quartile 1–quartile 3).

**Table 2:** Clinical and demographical data of cohort 2.

	**RoPE 0–5 (*n* = 14)**	**RoPE 6–10 (*n* = 24)**	***P*** **-value**
Age (years)	55 (53–60)	48 (40–54)	< 0.001
Sex (female)	4 (29%)	11 (46%)	0.329
Arterial hypertension	9 (64%)	5 (21%)	0.014
Diabetes	2 (14%)	1 (4%)	0.542
Adiposity	6 (43%)	5 (21%)	0.266
Dyslipoproteinemia	7 (50%)	10 (42%)	0.740
Peripheral artery disease	0 (0%)	0 (0%)	0.999
Nicotine consumption	10 (71%)	16 (67%)	0.999
Alcohol consumption	1 (7%)	2 (8%)	0.999
Coronary artery disease	0 (0%)	0 (0%)	0.999
Angina pectoris	0 (0%)	0 (0%)	0.999
Myocardial infarction	0 (0%)	0 (0%)	0.999
Atrial fibrillation	0 (0%)	0 (0%)	0.999
Pos. Family history	3 (21%)	7 (29%)	0.715
NIHSS at enrollment	0 (0–1.5)	0 (0–0)	0.161
mRS at enrollment	0 (0–1)	0 (0–0.75)	0.800
BMI (kg/m^2^)	27 (24–34)	25 (25–28)	0.212
CHA_2_DS_2_VASC	3.00 (2.75–4.00)	3.00 (2.00–3.00)	0.135
ESRS	2 (1,2)	1 (0.25–1)	0.003
Time from index event to follow-up (days)	98 (52–144)	115 (65–202)	0.560

Continuous data are presented as median (quartile 1–quartile 3).

**Figure 1:**
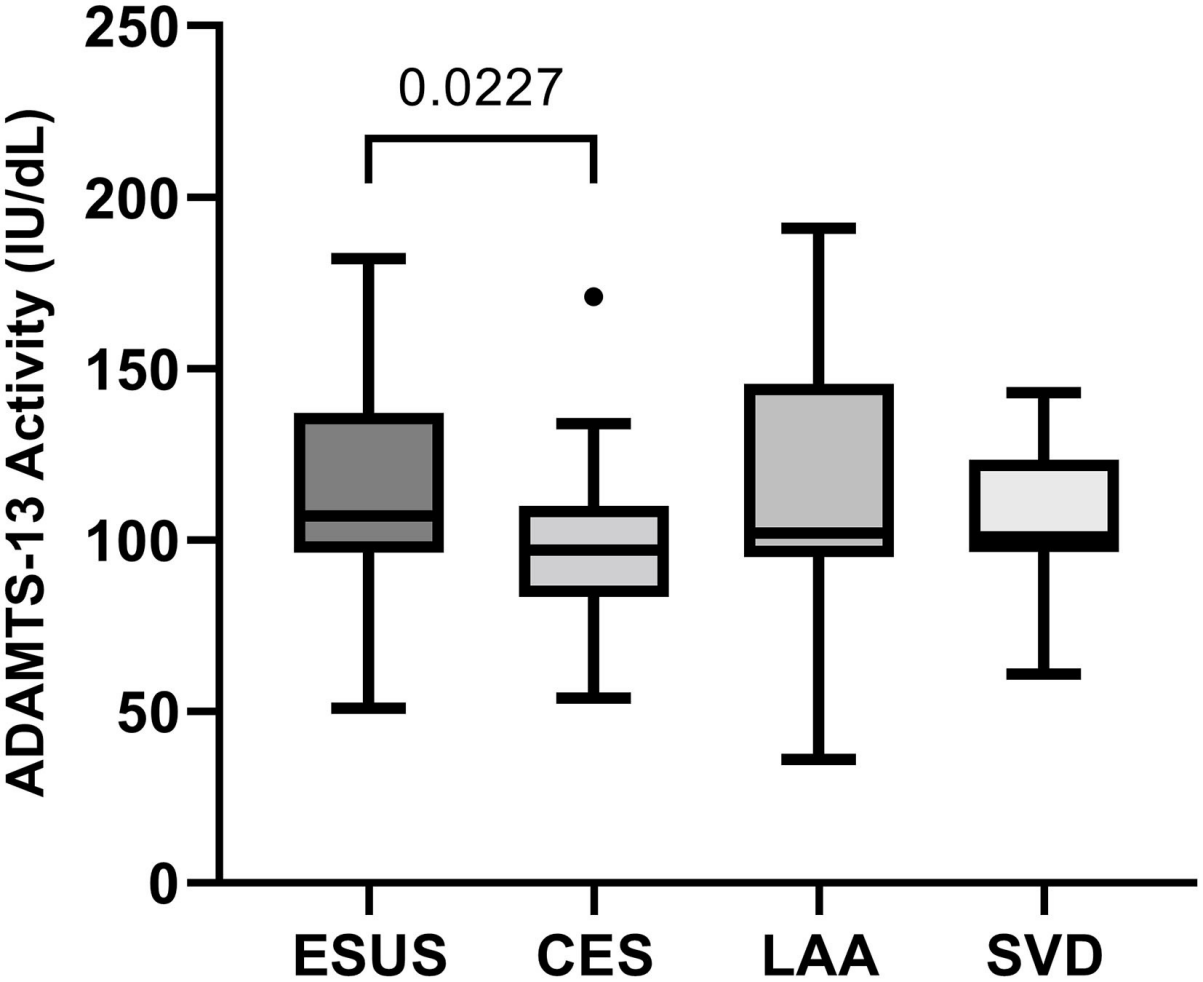
Comparison of ADAMTS-13 activity between stroke etiologies in cohort 1. CES, cardioembolic stroke due to atrial fibrillation; ESUS, embolic stroke of undetermined source; LAA, large-artery atherosclerosis; SVD, small vessel disease.

ADAMTS-13 activity was lower in patients with AF-related stroke compared to patients with ESUS (*p* = 0.023), while no other group differences were observed in the univariate analysis of cohort 1 (Figure 1). According to the logistic regression analysis adjusting for CHA_2_DS_2_VASC, the association between ADAMTS-13 and AF was shown to be confounded by vascular risk factors [unadjusted odds ratio (OR) for higher vs. lower ADAMTS-13 activity (median cutoff: 103 IU/dL): 0.224 (95% CI: 0.073–0.686, *p* = 0.009); adjusted OR for higher vs. lower ADAMTS-13 activity (median cutoff: 103 IU/dL): 0.335 (95% CI: 0.102–1.098, *p* = 0.071)]. Including ADAMTS-13 activity thus only slightly improved the AUROC [0.761 (95% CI: 0.643–0.879)] in discriminating AF from ESUS compared to CHA_2_DS_2_VASC alone [AUC: 0.737 (95% CI: 0.620–0.854); ΔAUROC: 0.024 (95% CI: −0.035–0.082)].

**Figure 2:**
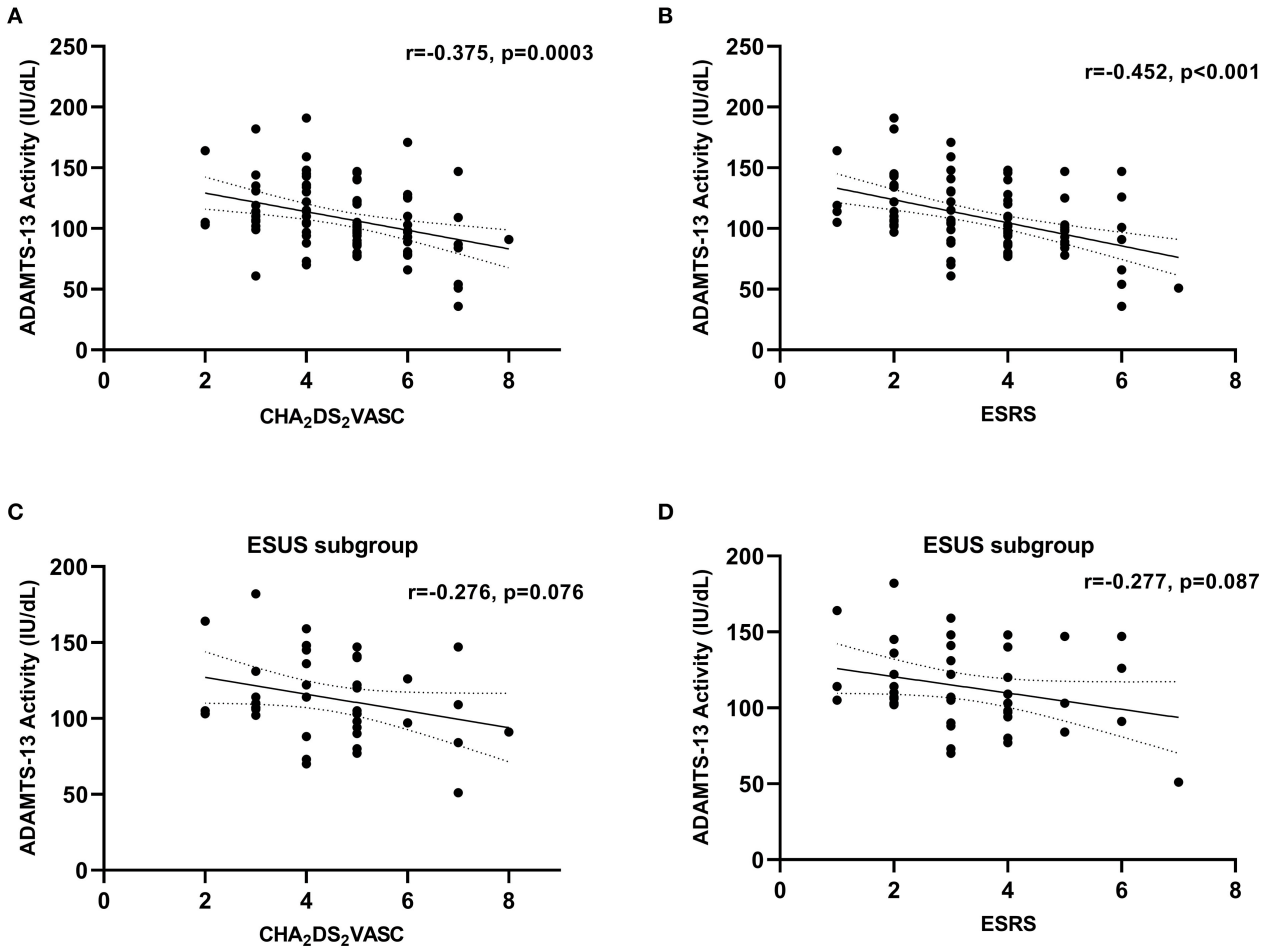
Correlation between ADAMTS-13 activity and vascular risk factors in cohort 1. **(A)** Whole cohort 1, CHA_2_DS_2_VASC; **(B)** whole cohort 1, ESRS; **(C)** ESUS subgroup, CHA_2_DS_2_VASC; **(D)** ESUS subgroup, ESRS. Correlation coefficients were calculated using Spearman's rank correlation.

For correlations between ADAMTS-13 activity and vascular risk scores in cohort 1, as stratified by stroke etiology, see Table 3, Figure 2. In all etiologies, inverse correlations could be identified with the largest effect sizes in the groups of CES and LAA. In patients with ESUS, the correlation coefficients were substantially lower (ESRS: *r* = −0.268, *p* = 0.087; and CHA_2_DS_2_VASC: *r* = −0.276, *p* = 0.076) than in patients with known stroke etiology (ESRS: *r* = −0.561, *p* < 0.001; and CHA_2_DS_2_VASC: *r* = −0.425, *p* = 0.003).

**Table 3:** Correlation of ADAMTS-13 activity with vascular risk scores in cohort 1, stratified by stroke etiology.

	**All strokes**	**ESUS**	**CES**	**LAA**	**SVD**
ADAMTS-13 activity/ESRS	*r* = −0.452	*r* = −0.268	*r* = −0.525	*r* = −0.752	*r* = −0.474
	*p* < 0.001	*p* = 0.087	*p* = 0.014	*p* = 0.032	*p* = 0.055
ADAMTS-13 activity/CHA_2_DS_2_VASC	*r* = −0.375	*r* = −0.276	*r* = −0.318	*r* = −0.528	*r* = −0.256
	*p* < 0.001	*p* = 0.076	*p* = 0.159	*p* = 0.179	*p* = 0.321

Correlation was calculated using Spearman's rank correlation.

**Figure 3:**
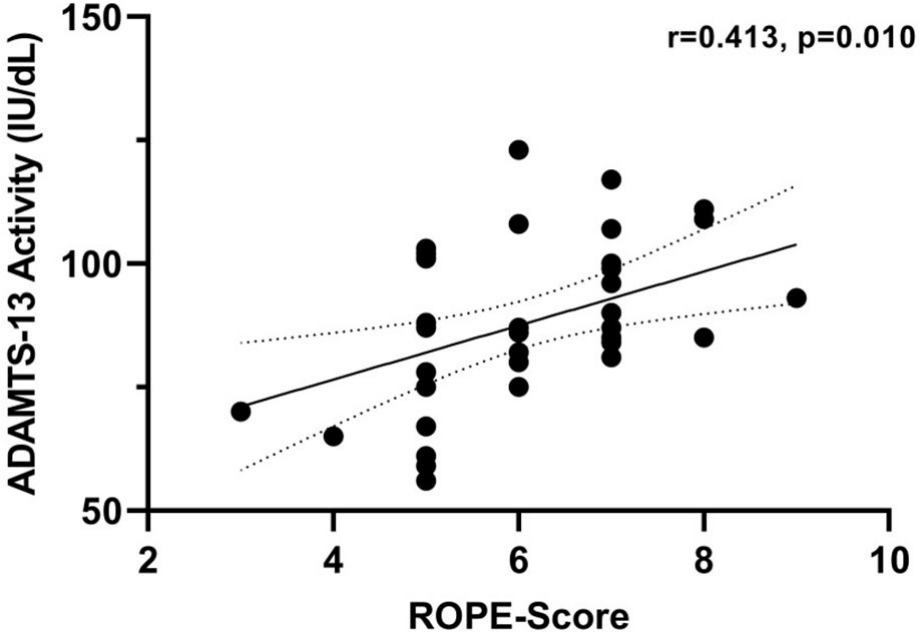
Correlation between ADAMTS-13 activity and RoPE-Score in cohort 2. Correlation coefficients were calculated using Spearman's rank correlation.

A comparison of patients in cohort 2 with a RoPE score of 0–5 vs. 6–10 revealed higher ADAMTS-13 activity in the latter group (*p* = 0.034). Accordingly, we found a positive correlation between ADAMTS-13 activity and the continuous RoPE score in cohort 2 (*r* = 0.413, *p* = 0.010, Figure 3). No correlation was found between ADAMTS-13 activity and ESRS (*r* = −0.107, *p* = 0.523), CHA_2_DS_2_VASC (*r* = 0.036, *p* = 0.829), or age (*r* = −0.240, *p* = 0.146) in cohort 2.

In addition, we analyzed which distinct risk factors may influence ADAMTS-13 activity in a linear regression model in both cohorts. As shown in Table 4, age, diabetes mellitus, a history of myocardial infarction, and nicotine consumption had the largest effects on lower ADAMTS-13 values. However, the precision of these estimates was quite low with the exception of age and diabetes mellitus.

**Table 4:** Multivariable linear regression models estimating the effect of distinct vascular risk factors on ADAMTS-13 activity in cohorts 1 and 2.

	**Cohort 1**	**Cohort 2**
	**β (95% confidence limits)**	**β (95% confidence limits)**
Age	−0.84 (−1.40; −0.287)	−0.63 (−1.33; 0.06)
Sex	2.22 (−9.95; 14.40)	0.10 (−11.18; 11.38)
Arterial hypertension	−1.33 (−16.69; 14.03)	0.61 (−12.45; 13.68)
Diabetes mellitus	−15.89 (−30.39; −1.39)	−6.97 (−27.69; 13.74)
Dyslipoproteinemia	−3.51 (−15.58; 8.56)	1.07 (−10.86; 13.00)
History of myocardial infarction	−19.72 (−44.85; 8.56)	N/A
Peripheral artery disease	−3.65 (−24.78; 17.49)	N/A
Adiposity	−3.94 (−19.43; 11.56)	2.55 (−10.06; 15.16)
Nicotine consumption	−7.56 (−19.87; 4.74)	−8.79 (−21.37; 3.79)

## Discussion

The clarification of stroke etiology has important implications for the choice of appropriate secondary stroke prevention. Despite all diagnostic efforts, the underlying mechanism remains cryptogenic in a substantial proportion of patients with stroke. Circulating mediators implicated in thrombosis may represent promising targets to uncover the individual mechanism of stroke or even to identify entirely novel causative pathways. Lower levels of ADAMTS-13 are associated with stroke (18, 29), myocardial infarction (29), and cardiovascular mortality (30). According to the results of a recent Mendelian randomization study, a lower ADAMTS-13 activity is, moreover, suggested to be causally linked to coronary heart disease and myocardial infarction (31).

Others and we have previously demonstrated that lower ADAMTS-13 activity was associated with an unfavorable clinical course following acute stroke therapies, that is, mechanical thrombectomy (32, 33) and intravenous thrombolysis (34, 35). Therefore, due to its potent thrombolytic activity, ADAMTS-13 may be regarded as a future therapeutic target in acute stroke (19, 36, 37). Despite the increasing evidence on the role of ADAMTS-13 in stroke pathophysiology, however, less is known regarding its potential as a biomarker of stroke etiology and as an indicator of cerebrovascular risk among specific stroke causes. Thus, we aimed to investigate ADAMTS-13 activity in two thoroughly characterized stroke cohorts with an emphasis on ESUS.

The identification of patients who are at high risk for AF is particularly relevant in light of the current evidence on ESUS (4). We, therefore, addressed the question of whether ADAMTS-13 could play a role as a biomarker for this purpose. Although the levels of ADAMTS-13 are significantly lower in patients with AF, the diagnostic additional value compared with vascular risk factors as subsumed with the CHA_2_DS_2_VASC score remains limited. In line with this, it was recently shown that the CHA_2_DS_2_VASC is already a sound predictor for the diagnosis of AF in patients with embolic stroke (28). Importantly, our analyses rather revealed that ADAMTS-13 activity is lower in patients with a higher burden of vascular risk factors, as determined by the CHA_2_DS_2_VASC and the ESRS. Both the CHA_2_DS_2_VASC and the ESRS are associated with risk for stroke recurrence, death, and other cardiovascular events also in patients without AF (38). However, the diagnostic accuracy for both scores was modest (38). Accordingly, future studies should further investigate the extent to which ADAMTS-13 may provide additional benefit in predicting recurrent vascular events in different stroke subtypes. This is again especially relevant for the ESUS population, as due to the high heterogeneity, there are still only a few reliable predictors to estimate individual hazards.

In this context, the subset of stroke patients with PFO represents a distinctive group (39). Because of the frequent prevalence of PFO in the general population, there is often doubt in patients with stroke about the contribution of a PFO to cerebral ischemia *via* the mechanism of paradoxical embolism, and it remains unclear why a large proportion of individuals with PFO never suffer a stroke. Clinical or imaging findings of DVT are found in only a small proportion of patients with cryptogenic stroke and PFO. According to a meta-analysis of randomized controlled trials, the re-infarction rate in patients with a PFO as the probable cause is only 1% under the best medical treatment (40). The RoPE score was developed to identify stroke patients with PFO who are at high risk for a mechanism *via* paradoxical embolism (27). Indeed, it was recently shown that patients with a higher risk for stroke-related PFO, that is, with higher RoPE scores, are more likely to benefit from PFO closure in terms of secondary stroke prevention than patients with lower RoPE scores, indicating that the RoPE score actually can identify pathogenic PFOs (41). Studies published in 2017 with extended follow-up of patients with stroke aged < 60 years finally demonstrated a preventive effect of PFO closure after cryptogenic stroke (42–44). However, the absolute effect size is relatively small, with a number needed to treat of 46.5 to prevent one event during 3.7 years (45). A novel biomarker that additionally assists individualized decision-making in this young and vulnerable stroke group would thus be of substantial value (39). Therefore, based on the previous evidence on the role of ADAMTS-13 in venous thrombosis, we hypothesized that patients with a higher probability for a pathogenic PFO may reveal lower ADAMTS-13 levels. Of note, this study is the first to address this question. In contrast to this hypothesis, we detected a positive correlation between the RoPE score and ADAMTS-13 activity in cohort 2, reflecting that with a higher burden of vascular risk factors, that is, lower RoPE scores, ADAMTS-13 activity declines. Interestingly, this association was not confirmed in studying the correlation between ADAMTS-13 and ESRS or CHA_2_DS_2_VASC, which is likely due to the generally lower burden of vascular risk factors in cohort 2. In accordance with our findings, Bongers et al. showed that patients in a comparable age group such as cohort 2, who suffered cardiovascular disorders including stroke, had lower ADAMTS-13 levels than controls (46). Of note, it was reported that ADAMTS-13 can be inversely related to age (18). Thus, we were interested in whether the correlation between ADAMTS-13 activity and the RoPE score, which largely depends on age categories, might be driven by age-dependent effects. Interestingly, this could not be confirmed according to the correlation analysis. Overall, our results indicate that ADAMTS-13 is associated with vascular risk factors and not with the risk of paradoxical embolism in the young collective of patients with PFO-associated stroke.

Of note, according to the multivariable linear regression analysis, age, diabetes mellitus, a history of myocardial infarction, and nicotine consumption were likely to have the largest effects on lower ADAMTS-13 activity in both cohorts. However, due to the small sample sizes, this finding must be interpreted as hypothesis-generating and needs validation in larger cohorts of patients with stroke.

This study has some further limitations. As described earlier, this study provides the first data on ADAMTS-13 levels in patients with ESUS and particularly in stroke patients with PFO. Our results thus need to be confirmed by further studies. In this course, longitudinal observation regarding vascular endpoints is also warranted to investigate whether ADAMTS-13 may serve as a predictive biomarker of stroke recurrence in patients with known and unknown etiologies. Furthermore, it would be of particular interest to show whether there are etiological differences in ADAMTS-13 activity also in the acute phase following stroke. Strengths of our study include the utilization of two prospectively enrolled and comprehensively characterized stroke cohorts with biomarker sampling in the long-term course, which excludes stroke-induced effects on ADAMTS-13 levels.

In conclusion, in the long-term course after stroke due to different etiologies, ADAMTS-13 activity inversely correlates with the number of vascular risk factors. Further study is warranted to elucidate the significance of ADAMTS-13 as a mediator of cerebrovascular risk and as a potential diagnostic and therapeutic target.

## Data availability statement

The raw data supporting the conclusions of this article will be made available by the authors, without undue reservation.

## Ethics statement

The studies involving human participants were reviewed and approved by Ethics Committe at Hannover Medical School. The patients/participants provided their written informed consent to participate in this study.

## Author contributions

Conceptualization: GG, AL, J-TS, UBa, JW, and KW. Data curation, formal analysis, and supervision: GG, AL, and KW. Funding acquisition and validation: GG and KW. Investigation: GG, AL, J-TS, UBu, NZ, SB, MG, JE, RS, UBa, JW, and KW. Methodology: GG, UBu, RL, and KW. Project administration and writing-original draft: GG and AL. Visualization: GG. Writing—review and editing: J-TS, UBu, NZ, SB, MG, JE, RS, RL, UBa, JW, and KW. All authors contributed to the article and approved the submitted version.
